# Giant IgG4-Related Pseudotumor of the Esophagus Resected with Endoscopic Submucosal Dissection: A Case Report and Review of the Literature

**DOI:** 10.5146/tjpath.2020.01515

**Published:** 2021-09-15

**Authors:** Nese Ekinci, Duygu Unal Kocabey, Eylul Gun, Fatih Aslan

**Affiliations:** Department of Pathology, Izmir Katip Celebi University, Ataturk Training and Research Hospital, Izmir, Turkey; Department of Gastroenterology, Koc University Hospital, Istanbul, Turkey

**Keywords:** IgG4, IgG4-related disease, Esophagus, Endoscopic submucosal dissection, Autoimmune

## Abstract

IgG4-related disease (IgG4-RD) is a systemic autoimmune disorder that has been defined in various organs. The disease is characterized by typical clinicopathological features including a dense lymphoplasmacytic infiltrate rich in IgG4 positive plasma cells, storiform fibrosis, obliterative phlebitis, and often an elevated serum IgG4 level. Esophageal IgG4-RD is rare, and its presentation as a solid mass is even more rare. Only 15 previous cases of IgG4-related esophageal disease have been described. We herein present a case of giant IgG4-related pseudotumor of the esophagus resected with endoscopic submucosal dissection (ESD) and a review of the literature. The patient was a 67-year-old man who was admitted to our hospital for assessment of progressive dysphagia. Upper gastrointestinal endoscopy revealed a 9 cm mass in the cervical esophagus. In the previous two hospitals, the patient’s mass could not be diagnosed despite repeated biopsies. Because of concerns regarding malignancy, endoscopic submucosal dissection was performed. Histopathological examination showed dense lymphoplasmacytic infiltration with predominant IgG4-positive plasma cells on a sclerotic background. The patient was diagnosed with IgG4-RD. During the follow-up, no residual mass was detected but the patient was diagnosed with lung adenocarcinoma. We present a unique case of giant IgG4-related pseudotumor of the esophagus. Resection with ESD of such a big mass of IgG4-RD in the esophageal region has never been reported before in the literature.

## INTRODUCTION

IgG4-related disease (IgG4-RD), originally described in the pancreas by Sarles et al. in 1961 (1), is a fascinating clinical entity of unknown etiology characterized by high serum IgG4 concentrations and tumefaction or tissue infiltration by IgG4-positive plasma cells. The disorder is recognized as a systemic disease characterized by specific histopathological findings that include the presence of predominant IgG4-positive plasma cells in a background of intense lymphoplasmacytic infiltration, storiform fibrosis, and obliterative phlebitis (2). It has been reported in almost any organ and tissue in the body including the pancreatobiliary system, lacrimal glands, salivary glands, central nervous system, thyroid, lungs, liver, gastrointestinal tract, kidney, prostate, retroperitoneum, arteries, lymph nodes, skin, and breast (3–5). The clinical presentation depends on the site of involvement (6). Involvement of the esophagus is extremely rare. Sometimes esophageal IgG4-RD may mimic malignancy both clinically and radiologically, especially when it forms a mass (7–9). We herein present a unique case of giant IgG4-related pseudotumor of the esophagus resected with endoscopic submucosal dissection (ESD) and a review of the literature.

## CASE REPORT

A 67-year-old Caucasian man presented with progressive dysphagia to solids over the last 2 years. His past medical history was uneventful. He had no other autoimmune diseases. He was an ex-smoker with a smoking history of 43 pack-years. The patient had previously visited 2 different hospitals, where he underwent upper gastrointestinal endoscopy, neck computed tomography (CT), and FDG-positron emission tomography (PET) because of the suspicion of malignancy. Upper gastrointestinal endoscopy revealed a mass-forming lesion prolapsing into the lumen of the cervical esophagus. The neck CT demonstrated a suspicion of gastrointestinal stromal tumor (GIST) and no concomitant lymphadenopathy. FDG-PET showed that the SUVmax of the mass was 10.0. Because of the suspicion of esophageal cancer, multiple biopsies were taken from the mass. However, the biopsy specimens were evaluated as squamous epithelial hyperplasia and inflammatory granulation tissue histologically which did not explain the tumoral nature of the lesion.

**Figure 1:**
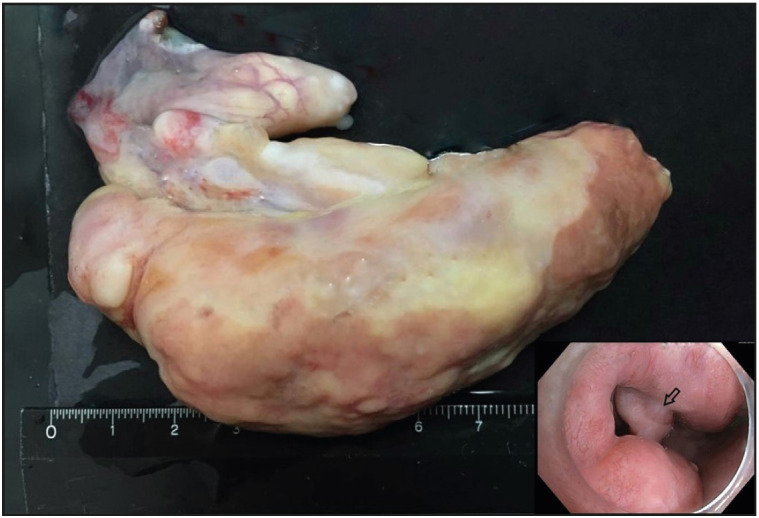
Macroscopic examination of the resected specimen revealed a polypoid tumor that measured 9x7x5 cm with mucosal surface erosion. Inset: Endoscopic view of upper esophagus demonstrated intraluminal mass (arrow) partially obstructing the esophageal lumen.

The patient presented to the gastroenterology department of our hospital. The physical examination was unremarkable. All laboratory data were within normal limits. Upper gastrointestinal endoscopy revealed an approximately 9 cm polypoid tumor partially obstructing the esophageal lumen, located about 15 cm from the incisors (Figure 1). Because of the obstruction and the concerns regarding malignancy, endoscopic removal of the lesion was planned.

He underwent a successful ESD. After marking the lesion, a mixture of saline, indigocarmin and hyaluronic acid was locally injected into the submucosal space to elevate the lesion. The borders were marked circumferentially using a needle knife tip (DualKnifeTM; Olympus, Tokyo, Japan) and careful dissection of the lateral borders and submucosal space was performed. En bloc removal of the lesion was performed successfully and it was sent to the pathology department for histological evaluation. Cauterization for bleeding was accomplished and enteral feeding was started in order to allow the esophageal mucosa to heal.

**Figure 2:**
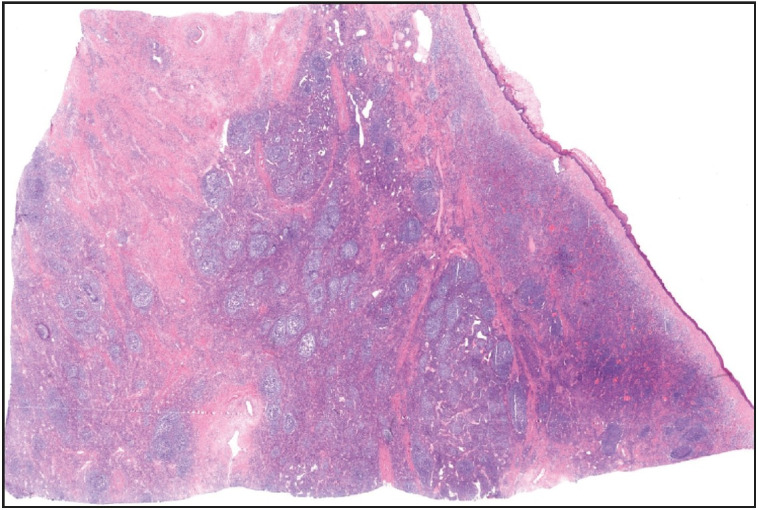
Low-power view shows dense lymphoplasmacytic inflammation and lymphoid follicles with prominent germinal centers on a sclerotic background beneath the squamous epithelium (H&E; x20).

**Figure 3:**
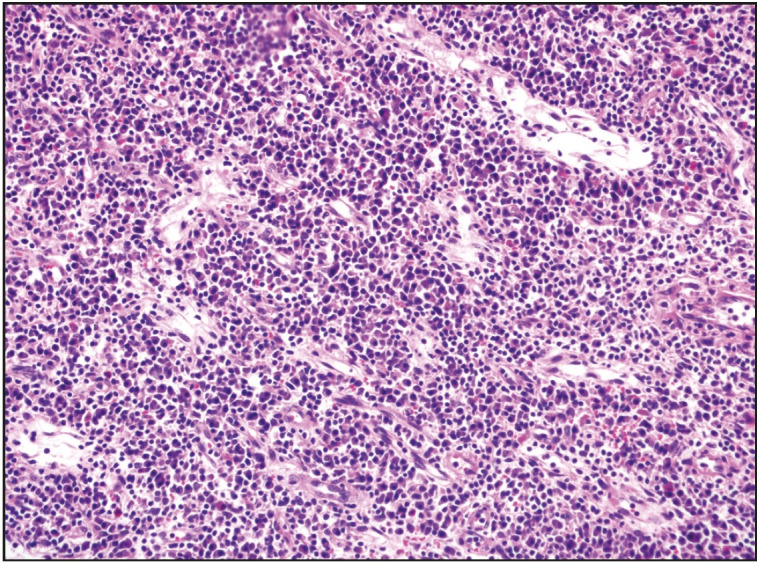
Diffuse infiltration of plasma cells (H&E; x200).

**Figure 4:**
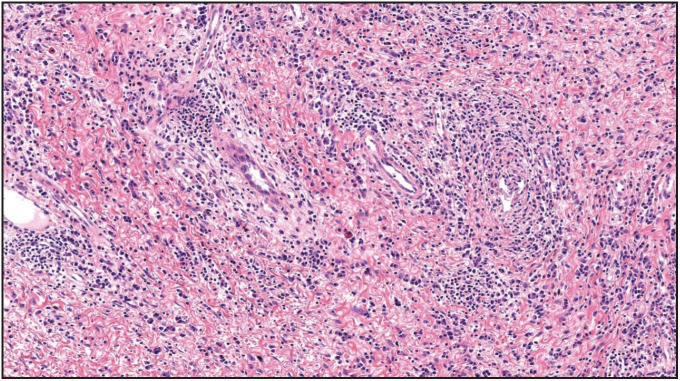
The stromal sclerosis resembling storiform fibrosis (H&E; x100).

**Figure 5:**
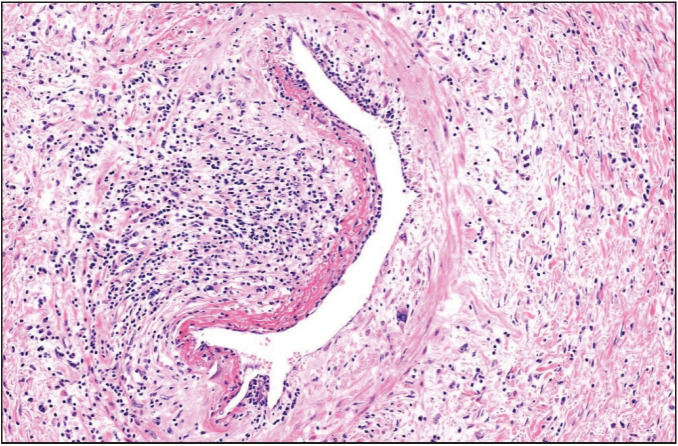
The blood vessel showing partial luminal obliteration by aggregated inflammatory cell infiltration (H&E; x100).

**Figure 6:**
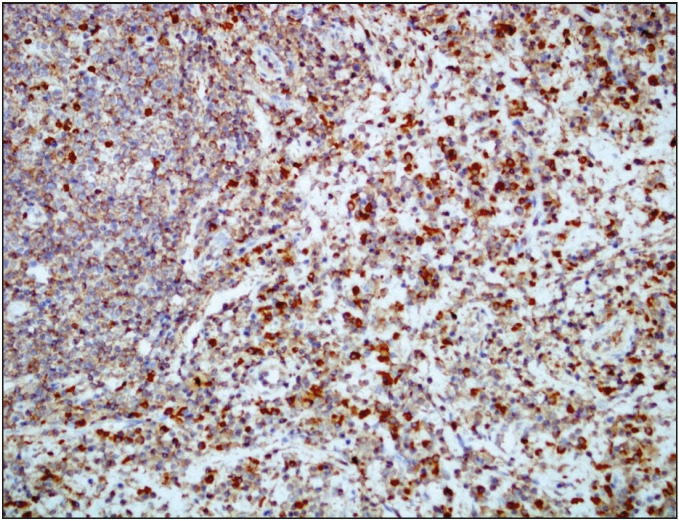
Dense infiltration of IgG4-positive plasma cells (IHC; x200).

Gross examination of the resected specimen revealed a polypoid tumor that measured 9x7x5 cm with mucosal surface erosion and a tan-white, solid, partially rubbery cut surface (Figure 1). Histological examination showed dense lymphoplasmacytic inflammation, lymphoid follicles with prominent germinal centers, and proliferation of small vessels on a sclerotic background beneath the ulcerated squamous epithelium (Figure 2). The inflammation consisted of abundant plasma cells admixed with some eosinophils (Figure 3). Stromal sclerosis resembling storiform fibrosis that is characterized by a whorled pattern of fibrosis was observed (Figure 4). Blood vessels showed partially luminal obliteration by aggregated inflammatory cell infiltration but there was no manifest obliterative phlebitis (Figure 5). There were reactive mucosal changes adjacent to the ulcer. Immunohistochemically, cytokeratin AE1/AE3, CD117, ALK, CD3, CD20, bcl2, CD10, CD38, kappa, lambda, IgG and IgG4 stains were performed. Stains for cytokeratin AE1/AE3, CD117, ALK were all negative in the lesion. A possible lymphoproliferative neoplasm was excluded by CD3, CD20, bcl2 and CD10 staining. CD38 revealed abundant plasma cells demonstrating a polyclonal staining pattern including the expression of both kappa and lambda. IgG4 immunostaining revealed 60 IgG4+ plasma cells per high-power field (HPF) (Figure 6). The ratio of IgG4+/IgG+ plasma cells was > 40%.

With the help of histopathological findings raising concern for IgG4-RD, the serum IgG4 level was checked afterwards. Ten days after the ESD procedure, the patient’s serum IgG4 level was found to be elevated at 179 mg/dL (reference range, 0-125 mg/dL). Based on comprehensive diagnostic criteria for IgG4-RD (10), the patient was finally diagnosed with a IgG4-related pseudotumor of the esophagus. He was discharged from the hospital after an uneventful post-procedural course. A written consent form was obtained from the patient’s relatives.

The patient did not receive any medication including corticosteroid therapy after the ESD procedure. He underwent neck and thorax CT two months after the ESD procedure. Neck CT showed no residual mass but unexpectedly the thorax CT showed a solid mass with an irregular spiculated contour of 24 mm size in the perihilar area of the lower lobe in the left lung. FDG-PET showed that SUVmax of the mass was 14.68 and there were concomitant hypermetabolic lymph nodes in the mediastinum. Due to the suspicion of lung cancer, bronchoscopy was performed. However, the mass could not be sampled despite repeated bronchoscopies. Bone scintigraphy showed a lesion suspicious in terms of metastasis in the left iliac crest. Biopsy taken from the lesion was evaluated as adenocarcinoma favoring the lung as primary origin. The patient received chemotherapy but unfortunately, 21 months after the ESD procedure, he passed away with unknown cause, and no autopsy was performed.

## DISCUSSION

IgG4-RD is an immune-mediated fibroinflammatory condition that has been described in a variety of organs (11). According to the comprehensive diagnostic criteria for IgG4-RD (10), 3 criteria were proposed for a diagnosis of IgG4-RD: (1) involvement with diffuse/localized swelling or masses in single or multiple organs; (2) an elevated serum IgG4 concentration (≥135 mg/dl); (3) histopathological findings that include a ratio of IgG4+/IgG+ plasma cells > 40% and >10 IgG4+ plasma cells/HPF in addition to marked lymphoplasmacytic infiltration and fibrosis. A diagnosis of IgG4-RD is definite in patients who fulﬁll the comprehensive diagnostic criteria for IgG4-RD (10). As our case fulfilled all 3 criteria and was a solid pseudotumor, our patient was diagnosed as definite IgG4-related pseudotumor of the esophagus.

In IgG4-related pancreatitis, especially in resection specimens, it has been reported that >30 IgG4+ plasma cells per HPF have acceptable specificity, and >50 IgG4+ plasma cells per HPF is highly specific. However, it is emphasized that the appropriate cut-off point may vary from organ to organ (12).

In 2019, American College of Rheumatology/European League Against Rheumatism developed the classification criteria for IgG4-RD as validated in a wide cohort of patients (13). The classification criteria for IgG4-RD are divided into 4 steps that include entry criteria, exclusion criteria, inclusion criteria, and total inclusion points. These steps contain the details from clinical, serological, radiological, and pathological evaluations. However, to obtain a relatively homogeneous population of patients, the organs or sites such as esophagus that are involved only rarely in IgG4-RD were excluded from that study.

Esophageal involvement of IgG4-RD (IgG4-RDE) was uncommonly reported in the literature. To our knowledge only 15 cases (7–9,14–18) have been described as IgG4-RDE in the literature previously (Table 1). Previous reports indicated that IgG4-RDE is more frequent in males and occur from a young age to elderly patients. Clinical symptoms of IgG4-RDE primarily include dysphagia and weight loss and more rarely odynophagia, epigastric pain, and acid reflux. Three of the 15 cases presented with an intraluminal esophageal mass (7–9), while 1 patient had an esophageal nodule (14). Operated cases were all removed with esophagectomy or esophagogastrectomy (7,14–16).

**Table 1:** Summary of reported cases of IgG4-related esophagitis.

**Case no.**	**Sex**	**Age, years**	**Symptoms**	**Imaging findings**	**IgG4 cells/ HPF**	**IgG4/IgG ratio**	**Serum IgG4**	**Diagnostic modality**	**Outcome**	**Report**
1	Male	23	Dysphagia and weight loss	Intraluminal mass and esophageal stricture	75	NA	NA	Esophagectomy	Uneventful postoperative course	Lopes et al. (7)
2	Male	63	Dysphagia and weight loss	Diffuse friability, ulceration and irregular stricture	200	NA	138 mg/dL (RR: 6-121 mg/dL)	Esophagectomy	Uneventful postoperative course	Lee et al. (15)
3	Female	63	Odynophagia and dysphagia	Circumferential severe ulcerative esophagitis	30	NA	NA	EGD biopsy	Small response to prednisolone and Rituximab	Dumas-Campagna et al. (17)
4	Male	33	Dysphagia and weight loss	Intraluminal mass and esophageal stricture	12	NA	264 mg/dL (RR: < 135 mg/dL)	US-guided needle biopsy	Improved after prednisolone	Oh et al. (8)
5	Male	60	Acid reflux	Intraluminal mass	>50	>30%	1590 mg/L (RR: 80–1400 mg/L)	EGD biopsy	Partial response to steroid therapy	Yang et al. (9)
6	Female	76	Dysphagia and weight loss	Circumferential erosion and stricture	>10	>40%	9.8 mg/dL (RR: 4.8-105 mg/dL)	Esophagectomy	Uneventful postoperative course	Mori et al. (16)
7	Male	47	Dysphagia	Stricture with friable mucosa	110	60	Normal level	EGD biopsy	Improved after prednisolone and mycophenolate mofetil	Obiorah et al. (14)
8	Female	79	Dysphagia	Stricture with friable mucosa	70	50	Normal level	EGD biopsy	Improved after prednisolone and mycophenolate mofetil	Obiorah et al. (14)
9	Male	48	Dysphagia	Multiple rings and stricture	50	80	Normal level	EGD biopsy	NA	Obiorah et al. (14)
10	Male	63	Dysphagia	Dilated distal esophagus with erosive esophagitis	50	60	NA	EGD biopsy	Uneventful post Botox injections and steroid therapy	Obiorah et al. (14)
11	Male	18	Dysphagia	Unremarkable endoscopy	60	80	NA	EGD biopsy	NA	Obiorah et al. (14)
12	Male	67	Dysphagia	Esophageal nodule and esophagitis	50	90	NA	EGD biopsy	NA	Obiorah et al. (14)
13	Male	52	Dysphagia	Dilated distal esophagus and diverticulum	90	90	NA	Esophagogastrectomy	Postesophagectomy stricture	Obiorah et al. (14)
14	Male	58	Epigastric pain	Severe erosive esophagitis	50	60	NA	EGD biopsy	NA	Obiorah et al. (14)
15	Male	56	Dysphagia and weight loss	Stricture and mucosal abrasion	>50	>90%	171.0 mg/dL	Esophagectomy	Uneventful postoperative course and improved after prednisolone	Jang et al. (18)

**NA:** Not available, **RR:** Reference range, **EGD:** Esophagogastroduodenoscopy.

In previous reports, the size of the IgG4-RDE mass in cases with one ranged from 1.5 cm to 3.9 cm (7,8). The current case is the largest of IgG4-RDE cases with a mass in the literature. Furthermore, our case is the first one that was removed with a successful ESD.

In 2017, a series of IgG4-RDE cases that consisted of 8 subjects was compared with chronic esophagitis, not otherwise specified, by Obiorah et al. Storiform fibrosis and high-density lymphoplasmacytic infiltrate were present in all cases with IgG4-RDE, while obliterative phlebitis was found in only 3 of 8 cases (14). It was reported that the mean number of IgG4+ plasma cells/HPF in the cases with IgG4-RDE was 66.9 and the mean IgG4:IgG ratio was 0.76. Both the number of IgG4+ plasma cells/HPF and the ratio of IgG4:IgG were significantly higher thanin the cases with chronic esophagitis, not otherwise specified. However, the serum IgG4 level was measured in only 3 of the cases with IgG4-RDE and found to be normal.

Steroid therapy is an effective and primary choice for the treatment of IgG4-RD (19). In our case, successful en bloc resection of the tumor with ESD was performed, so that the precise diagnosis could be made. ESD is an effective treatment of choice for superficial neoplasms of the gastrointestinal system and associated with lower morbidity than the surgical alternative. Esophageal ESD is a difficult procedure owing to the narrow lumen and thinner wall of the esophagus (20). Furthermore, even though the procedure of ESD seems to be difficult to achieve in a lesion with a giant size of 9 cm as in our study, the endoscopists in our facility are highly skilled in this regard and therefore this lesion was successfully removed in one piece. This happens to be the first case to report the use of ESD for the treatment of a patient with an IgG4-related pseudotumor.

IgG4-RD is considered an autoimmune disorder, and malignancy is associated with the dysregulated immune system (21). Some cohorts of patients with IgG4-RD have proposed that a history of cancer may be associated with the development of IgG4-RD (22–24). In patients with IgG4-RD, the most common malignancies are prostate cancer and hematological malignancies (22,23). In our case, metastatic lung cancer was found during the follow up. Although the relationship between malignancy and the IgG4RD is unclear, there are some possible explanations. The explanations contain the triggering of malignancy by autoantigen expression leading to IgG4-RD, common risk factors, and genetic predispositions for both IgG4-RD and malignancy (22).

In conclusion, IgG4-RDE can present with a mass and may lead to a misdiagnosis of malignancy, both clinically and radiologically. Diagnosis of IgG4-RDE requires a careful sampling of the lesion in order to perform a correct histopathological examination. However, it may be difficult to diagnose IgG4-RDE with small endoscopic biopsy specimens in some patients. In cases of suspicion, the biopsy/resection specimens should be stained with IgG4 and IgG immunohistochemically and the serum IgG4 level of the patient should be checked. In patients with an esophageal mass, consideration of IgG4-RDE in the differential diagnosis by the clinicians and pathologists will ensure the correct diagnosis and prevent unnecessary overtreatment.

## Conflict of INTEREST

None of the authors have any conflicts of interest to declare.

## References

[ref-1] Sarles H., Sarles J. C., Muratore R., Guien C. (1961). Chronic inflammatory sclerosis of the pancreas--an autonomous pancreatic disease?. Am J Dig Dis.

[ref-2] Kamisawa Terumi, Zen Yoh, Pillai Shiv, Stone John H. (2015). IgG4-related disease. Lancet.

[ref-3] Bledsoe Jacob R., Della-Torre Emanuel, Rovati Lucrezia, Deshpande Vikram (2018). IgG4-related disease: review of the histopathologic features, differential diagnosis, and therapeutic approach. APMIS.

[ref-4] Umehara Hisanori, Okazaki Kazuichi, Nakamura Takuji, Satoh-Nakamura Tomomi, Nakajima Akio, Kawano Mitsuhiro, Mimori Tsuneyo, Chiba Tsutomu (2017). Current approach to the diagnosis of IgG4-related disease - Combination of comprehensive diagnostic and organ-specific criteria. Mod Rheumatol.

[ref-5] Notohara Kenji, Kamisawa Terumi, Uchida Kazushige, Zen Yoh, Kawano Mitsuhiro, Kasashima Satomi, Sato Yasuharu, Shiokawa Masahiro, Uehara Takeshi, Yoshifuji Hajime, Hayashi Hiroko, Inoue Koichi, Iwasaki Keisuke, Kawano Hiroo, Matsubayashi Hiroyuki, Moritani Yukitoshi, Murakawa Katsuhiko, Oka Yoshio, Tateno Masatoshi, Okazaki Kazuichi, Chiba Tsutomu (2018). Gastrointestinal manifestation of immunoglobulin G4-related disease: clarification through a multicenter survey. J Gastroenterol.

[ref-6] Divatia Mukul, Kim Sun A., Ro Jae Y. (2012). IgG4-related sclerosing disease, an emerging entity: a review of a multi-system disease. Yonsei Med J.

[ref-7] Lopes James, Hochwald Steven N., Lancia Nicholas, Dixon Lisa R., Ben-David Kfir (2010). Autoimmune esophagitis: IgG4-related tumors of the esophagus. J Gastrointest Surg.

[ref-8] Oh Ji Hyun, Lee Tae Hee, Kim Hyo Shik, Jung Chan Sung, Lee Joon Seong, Hong Su Jin, Jin So-Young (2015). Esophageal Involvement of Immunoglobulin G4-Related Disease: A Case Report and Literature Review. Medicine (Baltimore).

[ref-9] Yang Lang, Jin Peng, Sheng Jian-qiu (2015). Immunoglobulin G4-related disease (IgG4-RD) affecting the esophagus, stomach, and liver. Endoscopy.

[ref-10] Umehara Hisanori, Okazaki Kazuichi, Masaki Yasufumi, Kawano Mitsuhiro, Yamamoto Motohisa, Saeki Takako, Matsui Shoko, Yoshino Tadashi, Nakamura Shigeo, Kawa Shigeyuki, Hamano Hideaki, Kamisawa Terumi, Shimosegawa Toru, Shimatsu Akira, Nakamura Seiji, Ito Tetsuhide, Notohara Kenji, Sumida Takayuki, Tanaka Yoshiya, Mimori Tsuneyo, Chiba Tsutomu, Mishima Michiaki, Hibi Toshifumi, Tsubouchi Hirohito, Inui Kazuo, Ohara Hirotaka (2012). Comprehensive diagnostic criteria for IgG4-related disease (IgG4-RD), 2011. Mod Rheumatol.

[ref-11] Stone John H., Zen Yoh, Deshpande Vikram (2012). IgG4-related disease. N Engl J Med.

[ref-12] Deshpande Vikram, Zen Yoh, Chan John Kc, Yi Eunhee E., Sato Yasuharu, Yoshino Tadashi, Klöppel Günter, Heathcote J. Godfrey, Khosroshahi Arezou, Ferry Judith A., Aalberse Rob C., Bloch Donald B., Brugge William R., Bateman Adrian C., Carruthers Mollie N., Chari Suresh T., Cheuk Wah, Cornell Lynn D., Fernandez-Del Castillo Carlos, Forcione David G., Hamilos Daniel L., Kamisawa Terumi, Kasashima Satomi, Kawa Shigeyuki, Kawano Mitsuhiro, Lauwers Gregory Y., Masaki Yasufumi, Nakanuma Yasuni, Notohara Kenji, Okazaki Kazuichi, Ryu Ji Kon, Saeki Takako, Sahani Dushyant V., Smyrk Thomas C., Stone James R., Takahira Masayuki, Webster George J., Yamamoto Motohisa, Zamboni Giuseppe, Umehara Hisanori, Stone John H. (2012). Consensus statement on the pathology of IgG4-related disease. Mod Pathol.

[ref-13] Wallace Zachary S., Naden Ray P., Chari Suresh, Choi Hyon K., Della-Torre Emanuel, Dicaire Jean-Francois, Hart Phillip A., Inoue Dai, Kawano Mitsuhiro, Khosroshahi Arezou, Lanzillotta Marco, Okazaki Kazuichi, Perugino Cory A., Sharma Amita, Saeki Takako, Schleinitz Nicolas, Takahashi Naoki, Umehara Hisanori, Zen Yoh, Stone John H., Members of the ACR/EULAR IgG4-RD Classification Criteria Working Group (2020). The 2019 American College of Rheumatology/European League Against Rheumatism classification criteria for IgG4-related disease. Ann Rheum Dis.

[ref-14] Obiorah I., Hussain A., Palese C., Azumi N., Benjamin S., Ozdemirli M. (2017). IgG4-related disease involving the esophagus: a clinicopathological study. Dis Esophagus.

[ref-15] Lee Hyuk, Joo Mee, Song Tae Jun, Chang Sun Hee, Kim Hanseong, Kim Yeon Soo, Ryoo Ji Yoon (2011). IgG4-related sclerosing esophagitis: a case report. Gastrointest Endosc.

[ref-16] Mori Shigeo, Tahashi Yoshiya, Uchida Kazushige, Ikeura Tsukasa, Danbara Naoyuki, Wakamatsu Takahiro, Kusuda Takeo, Takahashi Yu, Yanagawa Masato, Matsushita Mitsunobu, Ohe Chisato, Michiura Taku, Inoue Kentaro, Kon Masanori, Okazaki Kazuichi (2017). Sclerosing Esophagitis with IgG4-positive Plasma Cell Infiltration. Intern Med.

[ref-17] Dumas-Campagna Myriam, Bouchard Simon, Soucy Genevieve, Bouin Mickael (2014). IgG4-Related Esophageal Disease Presenting as Esophagitis Dissecans Superficialis With Chronic Strictures. J Clin Med Res.

[ref-18] Jang Sung Wook, Jeon Min Ho, Shin Hyun Deok (2019). IgG4-Related Disease with Esophageal Involvement. Case Rep Gastroenterol.

[ref-19] Martínez-Valle Fernando, Fernández-Codina Andreu, Pinal-Fernández Iago, Orozco-Gálvez Olimpia, Vilardell-Tarrés Miquel (2017). IgG4-related disease: Evidence from six recent cohorts. Autoimmun Rev.

[ref-20] Oyama Tsuneo (2014). Esophageal ESD: technique and prevention of complications. Gastrointest Endosc Clin N Am.

[ref-21] Shah Ami A., Casciola-Rosen Livia, Rosen Antony (2015). Review: cancer-induced autoimmunity in the rheumatic diseases. Arthritis Rheumatol.

[ref-22] Wallace Zachary S., Wallace Carly J., Lu Na, Choi Hyon K., Stone John H. (2016). Association of IgG4-Related Disease With History of Malignancy. Arthritis Rheumatol.

[ref-23] Poo S. X., Tham C. S. W., Smith C., Lee J., Cairns T., Galliford J., Hamdulay S., Jacyna M., Levy J. B., McAdoo S. P., Roufosse C., Wernig F., Mason J. C., Pusey C. D., Tam F. W. K., Tomlinson J. a. P. (2019). IgG4-related disease in a multi-ethnic community: clinical characteristics and association with malignancy. QJM.

[ref-24] Bledsoe Jacob R., Wallace Zachary S., Stone John H., Deshpande Vikram, Ferry Judith A. (2018). Lymphomas in IgG4-related disease: clinicopathologic features in a Western population. Virchows Arch.

